# Evidence for a recombinant origin of HIV-1 Group M from genomic variation

**DOI:** 10.1093/ve/vey039

**Published:** 2019-01-22

**Authors:** Abayomi S Olabode, Mariano Avino, Garway T Ng, Faisal Abu-Sardanah, David W Dick, Art F Y Poon

**Affiliations:** 1 Department of Pathology & Laboratory Medicine, Western University, London, Ontario, Canada; 2 Department of Applied Mathematics, Western University, London, Ontario, Canada; 3 Department of Microbiology & Immunology, Western University, London, Ontario, Canada

**Keywords:** HIV-1, molecular clock, recombination, subtype diversity, network clustering, phylogenetics

## Abstract

Reconstructing the early dynamics of the HIV-1 pandemic can provide crucial insights into the socioeconomic drivers of emerging infectious diseases in human populations, including the roles of urbanization and transportation networks. Current evidence indicates that the global pandemic comprising almost entirely of HIV-1/M originated around the 1920s in central Africa. However, these estimates are based on molecular clock estimates that are assumed to apply uniformly across the virus genome. There is growing evidence that recombination has played a significant role in the early history of the HIV-1 pandemic, such that different regions of the HIV-1 genome have different evolutionary histories. In this study, we have conducted a dated-tip analysis of all near full-length HIV-1/M genome sequences that were published in the GenBank database. We used a sliding window approach similar to the ‘bootscanning’ method for detecting breakpoints in inter-subtype recombinant sequences. We found evidence of substantial variation in estimated root dates among windows, with an estimated mean time to the most recent common ancestor of 1922. Estimates were significantly autocorrelated, which was more consistent with an early recombination event than with stochastic error variation in phylogenetic reconstruction and dating analyses. A piecewise regression analysis supported the existence of at least one recombination breakpoint in the HIV-1/M genome with interval-specific means around 1929 and 1913, respectively. This analysis demonstrates that a sliding window approach can accommodate early recombination events outside the established nomenclature of HIV-1/M subtypes, although it is difficult to incorporate the earliest available samples due to their limited genome coverage.

## 1. Introduction

At present, nearly 37 million individuals are living with human immunodeficiency virus type 1 (HIV-1) worldwide (UNAIDS 2017). Reconstructing the early dynamics of the HIV-1 pandemic can provide crucial insights into the socioeconomic drivers of emerging infectious diseases in human populations, including the roles of urbanization and transportation networks (Faria et al. 2014). Current evidence indicates that the global pandemic comprising almost entirely of HIV-1 Group M (HIV-1/M) originated around the 1920s in central Africa (Faria et al. 2014), where it had already diversified into the major subtypes before spreading around the world to their present-day distribution (Worobey et al. 2008; Tebit and Arts 2011). Much of the support for this model on the origin of HIV-1/M is based on the analysis of contemporary sequence diversity in specific regions of the HIV-1 genome (Korber et al. 2000). Due to the rapid evolution of this retrovirus, genetic differences accumulate on a directly observable time scale, making it possible to estimate the rate of molecular evolution (molecular clock) from sequences that were sampled at different points in time (Rodrigo and Felsenstein 1999), even when they are separated by only a few decades. In practice, these ‘dated-tip’ analyses of HIV-1/M diversity often rely on a number of assumptions including an absence of recombination, such that the entire span of the multiple sequence alignment (MSA) can be related through a single phylogeny (Korber et al. 2000; Worobey et al. 2008; Faria et al. 2014). Previous studies have shown that recombination can skew estimates of the molecular clock (Schierup and Hein 2000) and thereby estimates of times to common ancestors. For example, the transfer of divergent sequence by recombination from another subtype could inflate molecular clock estimates within the affected interval of the genome.

HIV-1/M is subdivided into nine major subtypes (Hemelaar 2012) and at least ninety circulating recombinant forms (CRFs) (Rodgers et al. 2018), which are defined as recombinants between the recognized subtypes that have been observed in three or more persons who are not epidemiologically linked (Robertson et al. 2000). Some CRFs have become highly prevalent in specific regions of the world, such as CRF01_AE in southeast Asia and CRF02_AG in west Africa (Tebit and Arts 2011). Homologous recombination from template-switching events during reverse-transcription occur frequently during the replication cycle of HIV-1 (Negroni and Buc 2001) and estimates of the recombination rates *in vivo* are either on the same order of magnitude as the mutation rate (Neher and Leitner 2010) or more. Thus, new recombinant forms are constantly emerging and inter-subtype recombination has recently been estimated to have occurred in ∼20% of HIV-1 lineages over the last 30 years of the global pandemic (Ward et al. 2013). There is growing evidence that recombination has played a significant role in the early history of the HIV-1 pandemic. For instance, several groups have described novel recombinant forms that incorporate genomic fragments from highly divergent lineages that precede the proliferation of the currently recognized HIV-1/M subtypes (Vidal et al. 2009; Tongo, Dorfman, and Martin 2016; Villabona Arenas et al. 2017). These observations suggest that what we presently recognize as ‘pure’ subtypes are quite likely recombinants of old, rare or extinct lineages, as illustrated by CRF01_AE and its extinct parental lineage that was originally designated Subtype E (Gao et al. 1996).

In this study, we have conducted a dated-tip analysis of all near full-length HIV-1/M genome sequences published in the GenBank database. We used a sliding window approach similar to the ‘bootscanning’ method for detecting breakpoints in inter-subtype recombinant sequences (Salminen et al. 1995) where a series of phylogenies are reconstructed on short intervals of the MSA. Instead of finding breakpoints, however, we use dated-tip methods to locate the root in each phylogeny and then use recently developed maximum-likelihood (ML) methods (To et al. 2016) to rescale the tree in chronological time. The intended purpose of this ‘rootscanning’ approach is to mitigate the effect of inter-subtype recombination on estimates of the molecular clock and the time to the most recent common ancestor (tMRCA). We describe a network-based strategy to overcome the inherent difficulties in generating a multiple alignment of divergent HIV-1 genome sequences that include extensive insertion-deletion (indel) variation. Our analysis is consistent with a recombinant origin of HIV-1/M diversity and supports a reassessment of the current HIV subtype nomenclature in the era of next-generation sequencing where full-length genomes are rapidly accumulating on a global scale (Pillay et al. 2015).

## 2. Methods

### 2.1 Data collection

We queried the GenBank nucleotide database using the search term ‘HIV-1’[porgn: txid11676] and limited the results to records with a minimum sequence length of 8,000 nt. This query yielded a total of 7,816 records on 11 October 2017. We manually screened these records for laboratory clones, *in vitro* recombinants, non-Group M variants and multiple samples from the same individual, in which case we retained only the earliest sample. For any sample with a missing collection date, we manually evaluated the publications associated with the GenBank record and restored the date information whenever possible; otherwise the record was discarded. After these steps, the resulting database comprised 3,900 records.

### 2.2 Sequence alignment and processing

Phylogenetic reconstruction and extrapolation of times to the most recent common ancestor requires the alignment of homologous nucleotides in the respective sequences. Using a standard MSA programs was not feasible for our purpose because the resulting alignment would contain an excessive number of gaps (see Supplementary Fig. S1), due to the extensive numbers of substitutions and indel events among sequences in HIV-1/M (that is, between defined subtypes and CRFs). Hence, we needed to filter the genome sequences for nucleotide sites retaining a relatively high degree of evolutionary homology. We adopted a pairwise alignment approach against a single reference genome sequence in which we systematically excised problematic sites. By removing any insertions relative to this modified reference, the resulting pairwise alignment will strictly retain only sites that are homologous to the reference.

First, we needed to generate an appropriate reference genome to carry out this Procrustean approach (Kern 2017) to sequence alignment. We generated a consensus sequence from a multiple alignment of a subset of genomes selected to be representative of the overall diversity in HIV-1/M. For this purpose, we used a p-spectrum kernel (Shawe-Taylor and Cristianini 2004) to generate a pairwise distance matrix from the entire set of unaligned genome sequences. Specifically, we used an in-house Python script to count the frequencies of all possible hexamers (p = 6) in each sequence and then calculated the inner product for every pair of frequency vectors:

$$k\left(s,t\right)=\underset{u\in A^{6}}{\Sigma }C\left(u,s\right)C\left(u,t\right)$$

where A = {*A*, *C*, *G*, *T*} and *C*(*x*, *y*) is a function that counts the number of instances of substring *x* in string *y*. We used the standard normalization such that 0 ≤*k*' ≤1 (Shawe-Taylor and Cristianini 2004):

$$k^{\prime }\left(s,t\right)=k\left(s,t\right)/\sqrt{k\left(s,s\right)k\left(t,t\right)}$$

The resulting similarity matrix was then converted into an undirected adjacency graph using a threshold of 0.93. We used the community detection algorithm called the *cluster_leading_eigen()* in the *igraph R* package to identify communities within the graph. For communities comprising greater than four members, one member having the highest degree centrality (connectivity) in each community was selected, giving rise to thirty-two sequences. Having a higher degree simply means that the sequence is highly connected with other sequences when compared with others (Anderson et al. 2012). The selected thirty-two representative sequences were aligned using MAFTT 9 version 7.271 (Katoh et al. 2002) to generate a MSA. We then generated a plurality-rule consensus sequence of 8,921 nt from the MSA. Next, we used an implementation of the Altschul-Erickson algorithm as a C extension for Python (https://github.com/ArtPoon/gotoh2) to generate pairwise alignments of all sequences against this consensus sequence, discarding any insertions relative to the consensus to yield a draft alignment. Finally, we generated a MSA of 10,171 nt from the draft alignment using MAFFT, then manually filtered residual regions with low homology (frequent indels) by removing alignment columns with a gap frequency >70%. This led to the removal of 1,544 columns to get a final alignment of 8,627 nt. To evaluate the impact of the number of genomes on run time and the length of the resulting alignment, we drew random samples of sequences without replacement to build sub-alignments ranging from 100 to 3,500 sequences at increments of 200 sequences, using MAFFT with twelve threads.

### 2.3 Phylogenetics and molecular clock analysis

Windows of 500 nt were extracted at steps of 100 nt from the final alignment leading to generation of eighty-two unique windows. Each window was visually inspected for residual alignment errors and problematic sites, which were manually removed. For each window, we reconstructed a ML phylogenetic tree using FastTree2 (Price, Dehal, and Arkin 2010) under the default general time-reversible model. Each tree was rooted based on the sample collection dates and a strict molecular clock model using the root-to-tip function (*rtt*) (Paradis, Claude, and Strimmer 2004) in the *ape* package in *R*. We used least-squares dating (LSD) (To et al. 2016) to rescale each rooted ML tree in time under a relaxed clock model, from which we estimated the tMRCA for each tree. In cases where the sample collection dates were reported at the reduced precision of years or months, we input the date for the corresponding tip as a range; for example, the entry ‘2009-06’ was input as ‘b(2009.414, 2009.493)’. To calculate the confidence intervals associated with the estimated tMRCA and rate, the LSD program uses a parametric bootstrap approach where the branch lengths in the tree are resampled from a Poisson distribution calibrated to the number of sites. We set the sampling number to 100 for this step. To characterize the autocorrelation in estimates of tMRCA across windows, we fit a piecewise linear regression model with two segments and zero slopes, and selected the best segment breakpoint using the Akaike information criterion. We measured for autocorrelation in the residuals of the regression analysis using the Durbin-Watson test as implemented in the *car* package (Lenth 2004) in *R*.

The Recombinant Identification Program (RIP) is an established method (Siepel et al. 1995) that was developed to detect inter-subtype recombination breakpoints in HIV-1/M sequences. However, RIP is a web-based program, making it difficult to perform batch processing on large numbers of full-length genome sequences. Therefore we developed an RIP-like script in Python to perform the analysis. First, we obtained a curated alignment of the HIV-1/M subtype reference sequences and the CRF CRF01_AE. For each query sequence, we used MAFFT to regenerate an MSA of the reference sequences with the query, and excluded all insertions in the query relative to the reference consensus. For sliding windows of 400 nt and a step size of 5 nt, we calculated the p-distances between the query and every reference, ignoring any gaps in either sequence, and tracked which references had the shortest and next-shortest distances. We used nonparametric bootstrap sampling (*n* = 200) to obtain a distribution of p-distances for the best reference, and rejected this reference if fewer than 90% of these distances were lower than the second-best reference. Finally, we used the change points in the resulting vector of reference names to identify potential breakpoints.

To corroborate our results from LSD, we analyzed the same data using a Bayesian sampling method implemented in BEAST (version 1.8.4) (Drummond and Rambaut 2007). Since it is not feasible to generate a random sample from the posterior distribution for trees relating to thousands of sequences, we uniformly down-sampled the sequence alignments in each window with respect to time, by selecting up to ten sequences per collection year at random without replacement. A previous simulation study (Hall, Woolhouse, and Rambaut 2016) determined that uniform sampling of sequences over time can provide unbiased reconstructions of past dynamics. This down-sampling resulted in a median of 294 sequences (range 284–294), which in our experience is at the upper limit of sample size at which a chain sample in BEAST might be expected to converge to the posterior distribution in a reasonable amount of time. We used a Python script to generate BEAST XML files from a model template for all eighty-two windows and ran the analyses in parallel on a local computing cluster. Based on previous work (Kinloch et al. 2016), the model template was configured to use the Tamura-Nei (TN93) nucleotide substitution model with rate variation approximated by a discretized gamma distribution with four rate categories, an uncorrelated lognormal molecular clock and a ‘skyline’ coalescent model (Drummond et al. 2006) with the default ten population size categories. Each chain sample was propagated for 10^8^ steps; the first 10^7^ steps were discarded as burn-in and the remainder was thinned at intervals of 10^4^ steps.

### 2.4 Selection analysis

We used the Fast Unconstrained Bayesian Approximation (FUBAR) method as implemented in HyPhy version 2.3.1 (Murrell et al. 2013) to identify codon sites undergoing either purifying or diversifying selection over the evolutionary history of HIV-1/M. FUBAR fits a limited number of rate categories defining non-synonymous (dN) and synonymous (dS) substitution rate combinations to individual codon sites for a given codon alignment and phylogenetic tree. We extracted codon alignments from the regions encoding *gag*, *pol* and *env* in our final sequence alignment obtained as described in the preceding Section 2.2. Because the reading frames of these major genes were disrupted by our filtering of extensive indel polymorphisms from the final alignment, we limited the codon alignments to cover a portion of each gene comparable to a single window in our dated-tip analysis. The resulting codon alignment each comprised 396 nt (132 codons) with the following consensus sequence coordinates: *gag* 793—1,197, *pol* 2,085—2,486, and *env* 6,225–6,623. Next, we used FastTree2 to reconstruct phylogenetic trees for each of the codon alignments.

## 3. Results

We retrieved 7,816 near full-length HIV-1 genome records from Genbank. After discarding irrelevant, incomplete or redundant sequences and restoring missing sample collection dates from publications associated with the respective records, we were left with 3,900 HIV-1/M genome sequences. Based on results from the SCUEAL subtyping program (Pond et al. 2009), 51% of the sequences were classified as ‘pure’ subtypes while 49% were classified as recombinant forms. The sequences were obtained from eighty-six different countries with a mean of 45 (range 1−460) sequences per country. The countries with the highest number of near full-length genome sequence records included United States (*n* = 1,752), Thailand (*n* = 719), South Africa (*n* = 698), China (*n* = 490), and Brazil (*n* = 414). The mean length of the genome sequences was 8,881 nt (range 8,000−9,897 nt). Sample collection dates associated with the sequences ranged between the years 1978 and 2016, with a mean of 108 (range 1−443) sequences per year.

**Figure 1.**
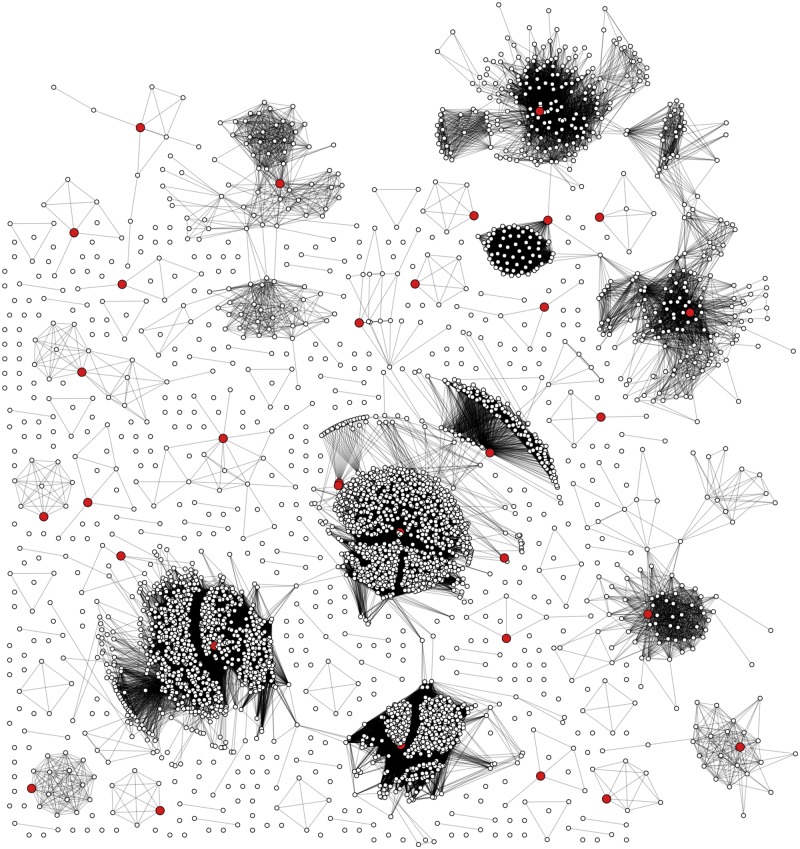
Network diagram representing the clustering of HIV-1 near full-length sequences into communities of genetically similar genomes. Each point (node) represents an HIV-1 genome sequence, and each connection (edge) indicates that the respective nodes have a pairwise *k*-mer distance below the threshold. Larger (red) nodes represent sequences with the maximum degree-size centrality in their respective network communities; these sequences were in a MSA to generate a draft consensus genome sequence.

Although it was feasible to construct a MSA from these data using a conventional program (e.g., MAFFT) in a reasonable amount of time (Supplementary Fig. S2), the resulting alignment became long and sparse due to an excessive number of gaps induced by highly divergent sequence intervals or non-homologous sequence insertions (Supplementary Fig. S3, top). Supplementary Fig. S1 summarizes the increasing alignment length with progressively larger numbers of genome sequences in the alignment, starting from a mean of 10,978 nt with 100 randomly selected sequences to a mean of 24,146 nt with 2,900 sequences. As a result, we constructed a consensus genome sequence from the multiple alignment of a subset of representative genomes, which we selected by a network clustering analysis of a p-spectrum kernel matrix (Shawe-Taylor and Cristianini 2004) of the entire data set. In brief, we calculated the inner products of hexamer frequencies for all pairs of genomes and converted the resulting matrix into a graph using a cutoff value to define edges. We used a clustering method to extract thirty-two subgraphs and identified the highest degree node in each subgraph. Figure 1 illustrates the entire graph with the identified representative nodes emerging from the thirty-two largest communities. Finally, we screened the MSA of genomes corresponding to these central nodes for regions of low homology and generated the consensus from the remaining sites. The consensus was used to make a pairwise draft alignment of all 3,900 genomes then, a final alignment was made using MAFFT (see Section 2 for details). The resulting alignment using this approach consisted mainly of regions of evolutionary homology across the genome sequences (Supplementary Fig. S3, bottom). When compared with the HXB2 reference sequences the *gag, pol*, *and env* genes of the consensus sequence are 1,475 (HXB2 1,503), 3,000 (HXB2 3,012), 2,507 (HXB2 2,571) nt in length, respectively.

### 3.1 Evolutionary history of HIV-1/M

**Figure 2.**
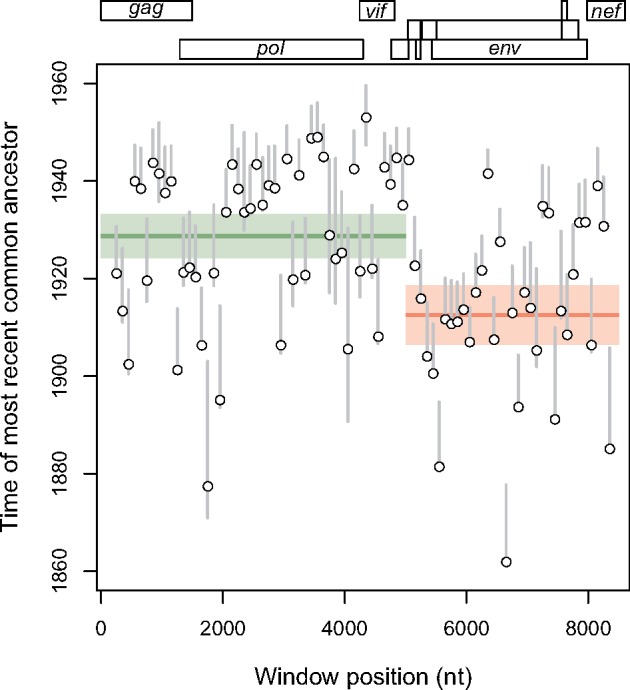
Origin of HIV-1/M based on LSD. The y-axis represents the estimated root dates (tMRCAs) for sequences in each window. The x-axis represents the genomic coordinates with each position corresponding to a gene region on the HIV-1 genome map at the top of the plot region. Each point represents data for a sliding window of 500 nt per sequence. The gray lines represent the 95% CI for each date estimate. The green and red lines indicate the breakpoint fragments computed using a piecewise linear regression model.

We employed a relaxed molecular clock analysis as implemented in the LSD program (To et al. 2016) to estimate the divergence times and substitution rate estimates across the HIV-1/M genomes. The estimated tMRCA from an analysis of the full-length HIV-1/M genome alignment was about 1,937 (95% CI 1,937.4−1,945.6). Given the inevitability of extensive recombination throughout the history of HIV-1/M, it is highly unlikely that a single phylogeny can adequately represent the evolutionary history of HIV-1/M. Thus, applying the same method to windows extracted from the genome alignment, we found evidence of substantial variation in tMRCA estimates among windows, with an estimated mean tMRCA of 1922 (Fig. 2). These estimates were significantly autocorrelated (Durbin-Watson Test, *P* < 0.05), which was more consistent with a genome-level effect than uncertainty in phylogenetic reconstruction and dating. Further, we show that the earlier estimates tended to be obtained from windows within the *env* gene region, which has frequently been targeted in studies of HIV-1 origins (Worobey et al. 2008; Faria et al. 2014).

**Figure 3.**
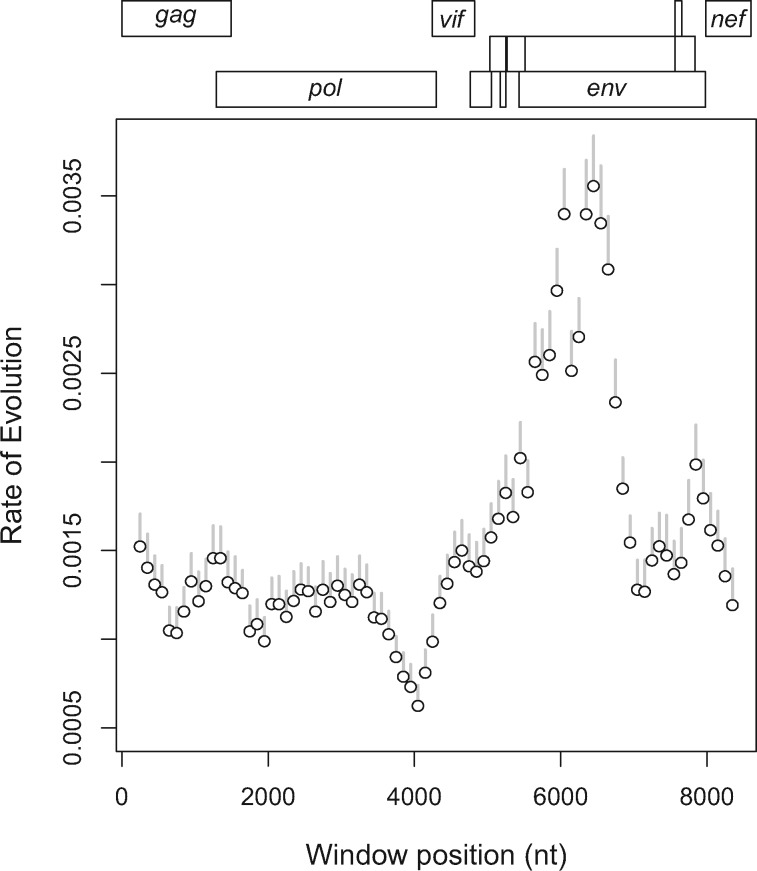
HIV-1/M substitution rate estimates based on LSD. The y-axis represents rates of substitution for sequences in each window. The x-axis represents the genomic coordinates with each position corresponding to a gene region on the HIV-1 genome map at the top. Each point represents data for a sliding window of 500 nt per sequence. The gray lines represent the 95% CI for each rate estimate.

Our results also demonstrate an appreciable degree of variation across windows for the estimated nucleotide substitution rates (Fig. 3). For the entire alignment, the LSD rate estimate was (95% CI). The regions with the highest substitution rate estimates generally coincided with the regions with earlier tMRCA estimates. The *env* gene region has been established as the most variable region across HIV-1 genomes (Leitner and Albert 1999). However, substitution rate estimates from the latter half of the *env* gene (about 7,000–8,000 in our coordinate system) were more similar to rate estimates obtained in *gag* or *pol*. We calculated the relationship between the alignment window and estimated root dates by fitting a piecewise linear regression model to the data. Using AIC-based model selection, the optimal piecewise model supported the existence of at least one deep recombination breakpoint in the HIV-1/M genome, with a mean tMRCA of about 1,929 (95% CI: 1,924−1,933) for the younger of two fragments and 1,913 (95% CI: 1,906−1,918) for the older fragment (Fig. 2).

**Figure 4.**
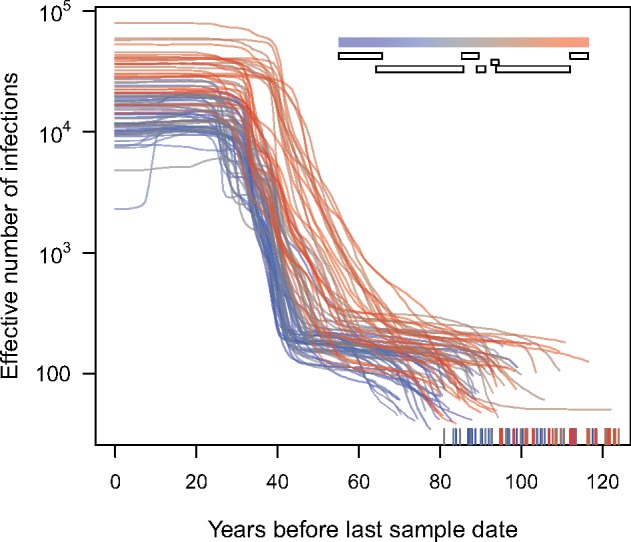
Bayesian skyline plots for all eighty-two windows in the HIV-1/M genome alignment. The vertical axis corresponds to mean estimates of the effective number of infections, which is roughly proportional to incidence. The horizontal axis represents the number of years prior to the most recent sample. Each trend line corresponds to a different window, whose location is indicated by color (see inset legend and genome diagram); in sum, the blue lines correspond to windows closer to the 5’ end of the genome, and red lines to windows closer to the 3’ end. Tickmarks along the horizontal axis represent the median estimate of the tMRCA for each window (see also Fig. 4).

In addition, we used BEAST to obtain a second set of estimates under a relaxed clock model for the times to the most recent common ancestor across the alignment windows. Since the number of sequences in each window was excessive for Bayesian sampling of phylogenies, we randomly sub-sampled the data uniformly across sample collection dates for roughly 300 sequences per window. Similar to our results from an LSD analysis of the entire data set, we found that the estimates from BEAST also tended to be earlier for windows in the 3’ half of the genome, including the region encoding HIV-1 *env* (Supplementary Fig. S4). We applied the same piecewise regression analysis with AIC model selection to identify a potential deep recombination breakpoint roughly in the same location as the previous analysis. Furthermore, reconstructions of the past dynamics of HIV-1/M based on the Bayesian skyline model (Fig. 4) reveal a distinct shift in dynamics between these two portions of the genome. Finally, we used an adaptation of the RIP method (Siepel et al. 1995) to detect putative inter-subtype recombination breakpoints in our genome alignment. Using this method, we detected breakpoints in 1,443 of 3,900 genome sequences, with a distinct bias for an even number of breakpoints per genome (Supplementary Fig. S5). We observed that the distribution of putative breakpoints was consistent with results from our piecewise regression analysis. Specifically, we observed that recombination breakpoints tended to accumulate around the 5’ end of the HIV-1 *env* gene (Supplementary Fig. S6), which suggested that this location of the genome is more tolerant of inter-subtype recombination.

### 3.2 Selection analysis

Purifying selection occurs when lineages carrying amino acid substitutions at particular sites have reduced fitness. Thus, purifying selection can be detected by comparing site-specific non-synonymous (dN or β) and synonymous (dS or α) substitution rates, where β − α < 0 indicates purifying selection, β – α ≍ 0 indicates neutral evolution, and β – α > 0 indicates diversifying selection (Yang et al. 2000). Purifying selection can skew dated-tip analyses because the removal of amino acid substitutions by selection causes the model to underestimate branch lengths, resulting in more recent estimates of the tMRCA. We observed substantial variation in estimates of β − α across subsets of codon sites corresponding to window alignments in the major genes of the HIV-1/M genome (*n* = 132 codons per gene, Supplementary Fig. S7). For example, the proportions of codon sites under significant (α_p_ = 0.05) purifying selection were 0.182, 0.121, and 0.265 for *env*, *gag*, and *pol*, respectively. However, we found no significant mean difference in β − α between *env* and the other two major genes (Wilcoxon test, *P* = 0.192).

## 4. Discussion

Although HIV-1 has a high effective rate of recombination (Neher and Leitner 2010) and inter-subtype recombination is prevalent (Ward et al. 2013), studies reconstructing the early history of the HIV-1/M pandemic have generally ruled out the potential effects of recombination as a simplifying assumption. There is growing evidence however that recombination has played an important role in the early evolution of HIV-1/M, and recombinant genomes have been documented between recognized HIV-1 subtypes and highly divergent unique HIV-1 lineages (Abecasis et al. 2007; Villabona Arenas et al. 2017). In addition, there has been growing controversy regarding the assignment of HIV-1 variants to a fixed nomenclature of pure subtypes and CRFs when our current understanding of the early and current global evolution of the virus at the genome-wide level may be incomplete (Abecasis et al. 2007; Tongo et al. 2015; Tongo, Dorfman, and Martin 2016, Zhang et al. 2010). Using a sliding window method on a highly curated multiple alignment of near full-length genomes, we found evidence of substantial variation in estimated root dates (tMRCAs) among windows. Across windows, the median date estimates averaged about 1922 (range 1861−1948), which is consistent with previous work that places the origin of HIV-1/M within the first three decades of the 20th century (Korber et al. 2000; Worobey et al. 2008; Faria et al. 2014). Our median tMRCA estimates were significantly autocorrelated along the genome, suggesting that the variation is not entirely explained by uncertainty in phylogenetic reconstruction or sampling variation. Instead, this pattern implies a deterministic effect of the region of the HIV-1 genome being analyzed on estimates of the tMRCA.

A significant influence of genomic regions on estimates of tMRCA can be explained by either recombination or selection, and it is possible that both processes have played some role. First, the observed pattern in tMRCA estimates may be the residual signature of at least one recombination event that occurred early in the evolutionary history of HIV-1/M, preceding the rapid expansion of this group from central Africa to the rest of the world. Put another way, the evolutionary history of HIV-1/M would be more accurately modeled by two phylogenies instead of one, where the phylogeny relating the 5’ portion of the genome has a more recent common ancestor. Our analysis of recombination breakpoints in the genome alignment implies that the regions flanking the *env* gene are more tolerant of inter-subtype recombination. This observation is consistent with a previous study of recombination hotspots in the HIV-1 genome (Archer et al. 2008), which postulated that selection favors the transfer of intact HIV-1 *env* between genomic backgrounds due to its particular importance in virus fitness. Second, this outcome may be the result of different levels of purifying selection in the HIV-1 genome (Wertheim and Kosakovsky Pond 2011). Purifying selection reduces the observable rate of molecular evolution by removing deleterious mutations at conserved sites (Ho et al. 2005). This greater constraint on tolerated substitutions shortens the time scale on which a molecular clock analysis is susceptible to saturation, where the number of substitutions between two sequences is so extreme that they become indistinguishable from completely unrelated sequences with respect to genetic distance. Although it is conceivable that diversifying selection might also drive sequences to saturation by accelerating substitution rates, with the effect that the apparent rate inferred from observed sequences is lower than the actual rate, this type of selection is generally limited to a minority of sites in coding sequences (Edwards et al. 2006; Wertheim and Kosakovsky Pond 2011). The HIV-1 *gag* and *pol* genes on the 5’ side of the genome tend to be more evolutionarily conserved than the *env* and *nef* genes that are encoded closer to the 3’ end (Alizon and Fraser 2013). Hence, the more recent tMRCA estimates from *gag* and *pol* are consistent with greater purifying selection filtering out mutations at conserved sites.

Here we enumerate the evidence from our study that supports an early recombination event as a more parsimonious explanation for the distribution of tMRCA estimates along the HIV-1 genome. First, the LSD estimates of the molecular clock we obtained from *nef* and the portion of *env* encoding gp41 were comparable to estimates from *gag* and *pol* (Fig. 3). We did not observe a concomitant increase in tMRCA estimates associated with gp41 and *nef* that would signify a similar effect of purifying selection in these regions. Second, our alignment largely excluded several major targets of diversifying selection, including the hypervariable loops in *env*, because these regions are problematic for generating a MSA across the global diversity of HIV-1/M. Indeed our analysis of site-specific selection (Supplementary Fig. S7) indicated that the majority of codon sites from the region of the filtered alignment corresponding to HIV-1 *env* were evolving under levels of purifying selection comparable to sites in *gag* and *pol*. No significant difference was found in the proportion of positively selected sites among these gene-specific windows. Third, the sliding window alignments do not exhibit a clear signature of saturation. For instance, Supplementary Fig. S8 plots the numbers of transitions and transversions between pairs of aligned sequences against a genetic distance corrected for multiple hits—a conventional method for detecting saturation (Xia and Lemey 2009)—for an arbitrary subset of window alignments, although this method does not rule out saturation at a minority of positively selected sites. This is consistent with recent work using a more complex time-calibrated approach, which did not find evidence of saturation in HIV-1 sequences (Duchêne, Ho, and Holmes 2015).

It is important to note that we only used data that were available in the public domain, and that our data collection does not represent a random sample of the global distribution of HIV-1/M. However, the primary objective was to sample the ancestors of HIV-1/M and our study does not draw any conclusions about the present global distribution of the virus. We postulate that recurrent introgression between countries of HIV-1 genomic variation through inter-subtype recombination limits the effect of sampling location, and that focusing the molecular clock analysis on relatively narrow windows of the genome instead amplifies the influence of sample collection dates. Our results support the hypothesis that the current global diversity of HIV-1/M may be a product of at least one recombination event between two or more ‘ancient’ variants, with an older fragment encoding the *env* and *nef* genes. It is tempting to speculate that an earlier origin of *env* may have been associated with the role of the envelope glycoproteins in host-specific adaptation (e.g. host cell attachment and entry as well as escape from neutralizing antibodies), but this hypothesis needs to be substantiated by further investigation. Our work demonstrates a novel approach to incorporating genome-scale variation in reconstructing the evolutionary history of HIV-1, instead of focusing on a specific region of the genome. We note that the sliding window approach does not eliminate the possibility of recombination events within each window; its purpose is to detect and accommodate early recombination events, deep in the phylogeny, that might otherwise skew estimates of the molecular clock. With growing availability of more cost-effective and rapid sequencing technologies and software tools for processing the resulting outputs, it is becoming more feasible to analyze the evolutionary history of HIV-1 on a genome-wide scale. In combination with appropriate phylogenetic tools, these data will inevitably advance our knowledge on the origin and evolution of HIV-1. For instance, we anticipate that the large-scale genome sequence analysis of HIV-1/M in Africa currently being conducted by the Phylogenetics and Networks for Generalized HIV Epidemics in Africa consortium (PANGEA-HIV) (Pillay et al. 2015) will deliver significant new insights into the evolution of this virus.

## Supplementary Material

Supplementary Figure S1

Supplementary Figure S2

Supplementary Figure S3

Supplementary Figure S4

Supplementary Figure S5

Supplementary Figure S6

Supplementary Figure S7

Supplementary Figure S8

Supplementary Data
