# Supplementary figures and images for: Evidence for a recombinant origin of HIV-1 Group M from genomic variation
Source: Virus Evol. 2019 Jan 22;5(1):vey039. doi: 10.1093/ve/vey039 (PMC6342232; doi:10.1093/ve/vey039)

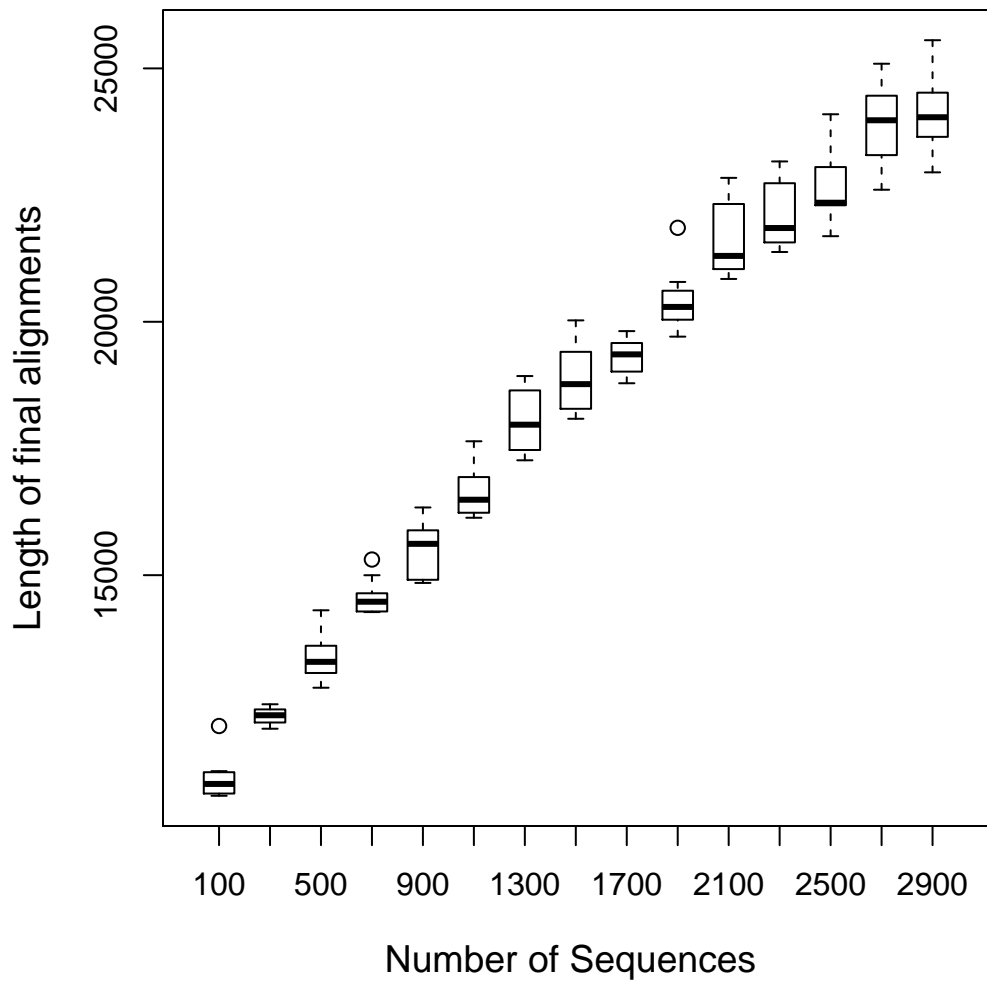

Supplement: Supplementary Figure S1 [file vey039_figure_s1.pdf]

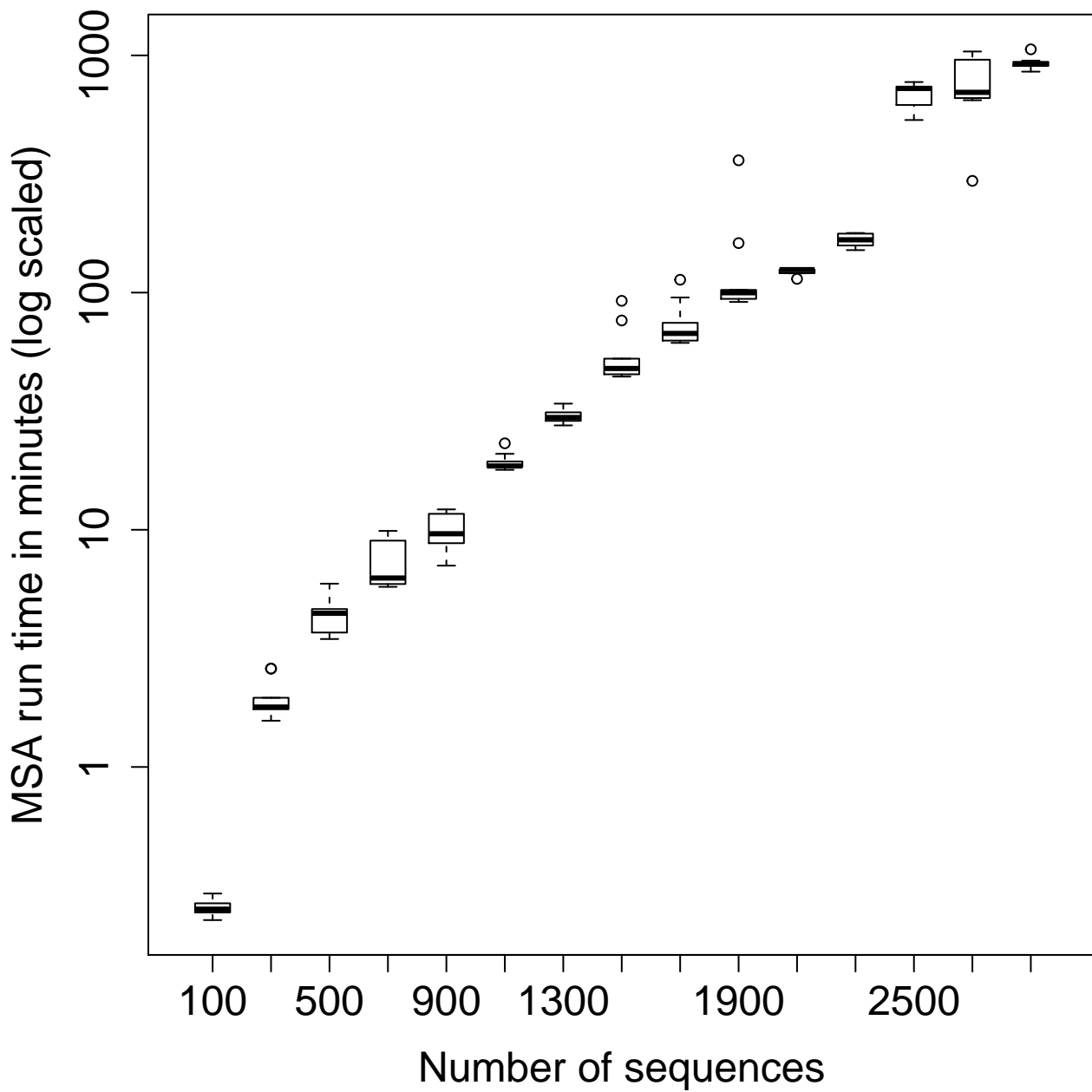

Supplement: Supplementary Figure S2 [file vey039_figure_s2.pdf]

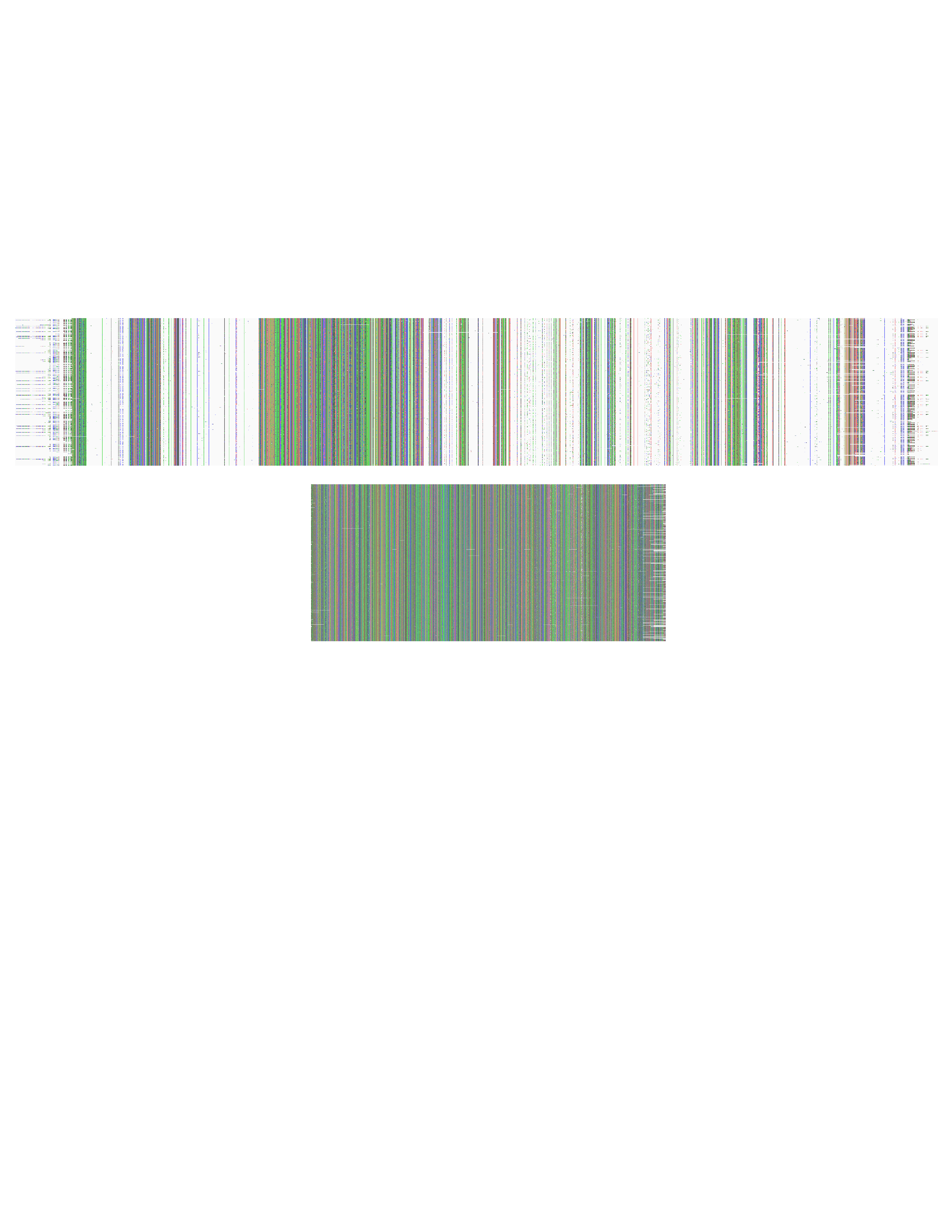

Supplement: Supplementary Figure S3 [file vey039_figure_s3.png]

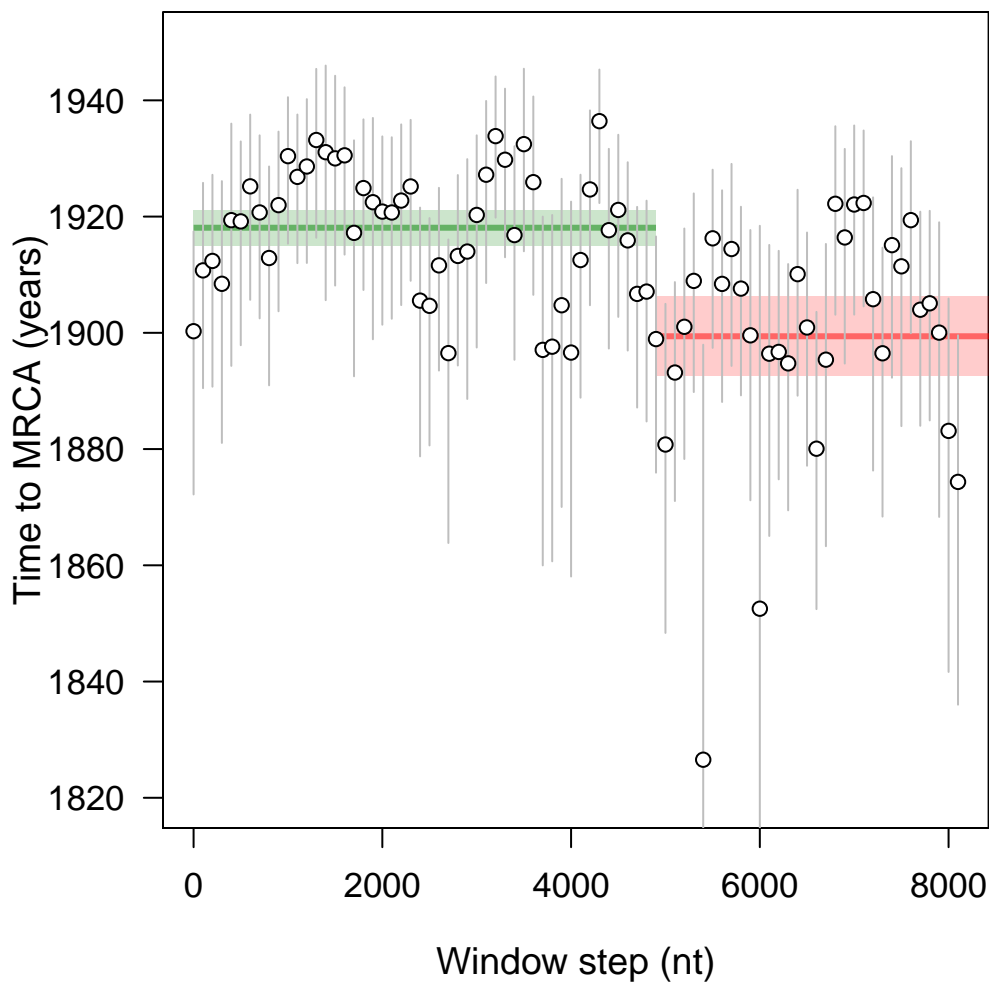

Supplement: Supplementary Figure S4 [file vey039_figure_s4.pdf]

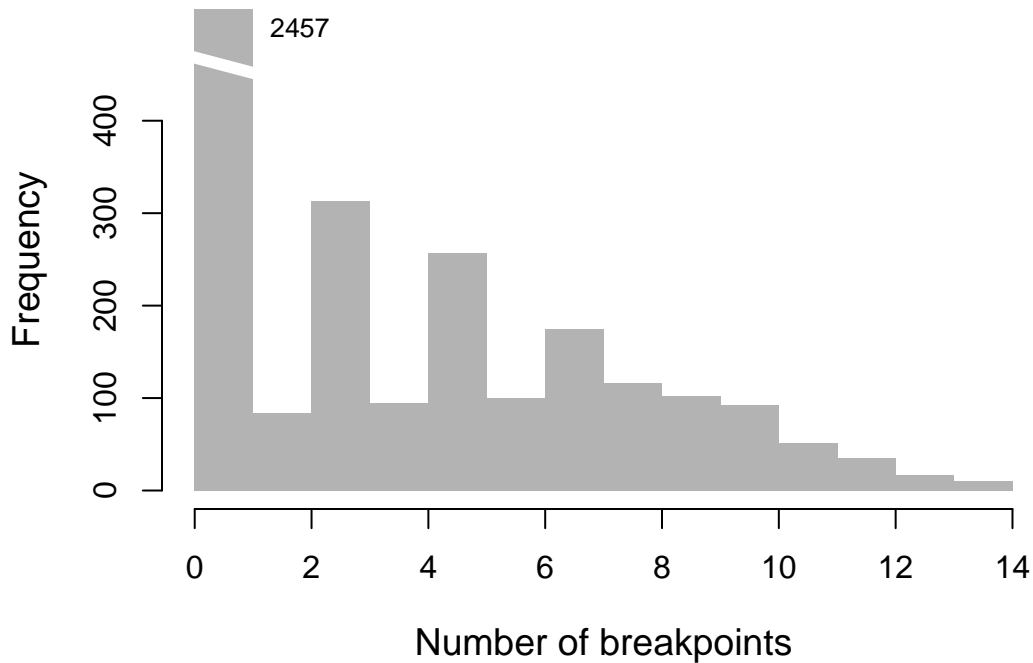

Supplement: Supplementary Figure S5 [file vey039_figure_s5.pdf]

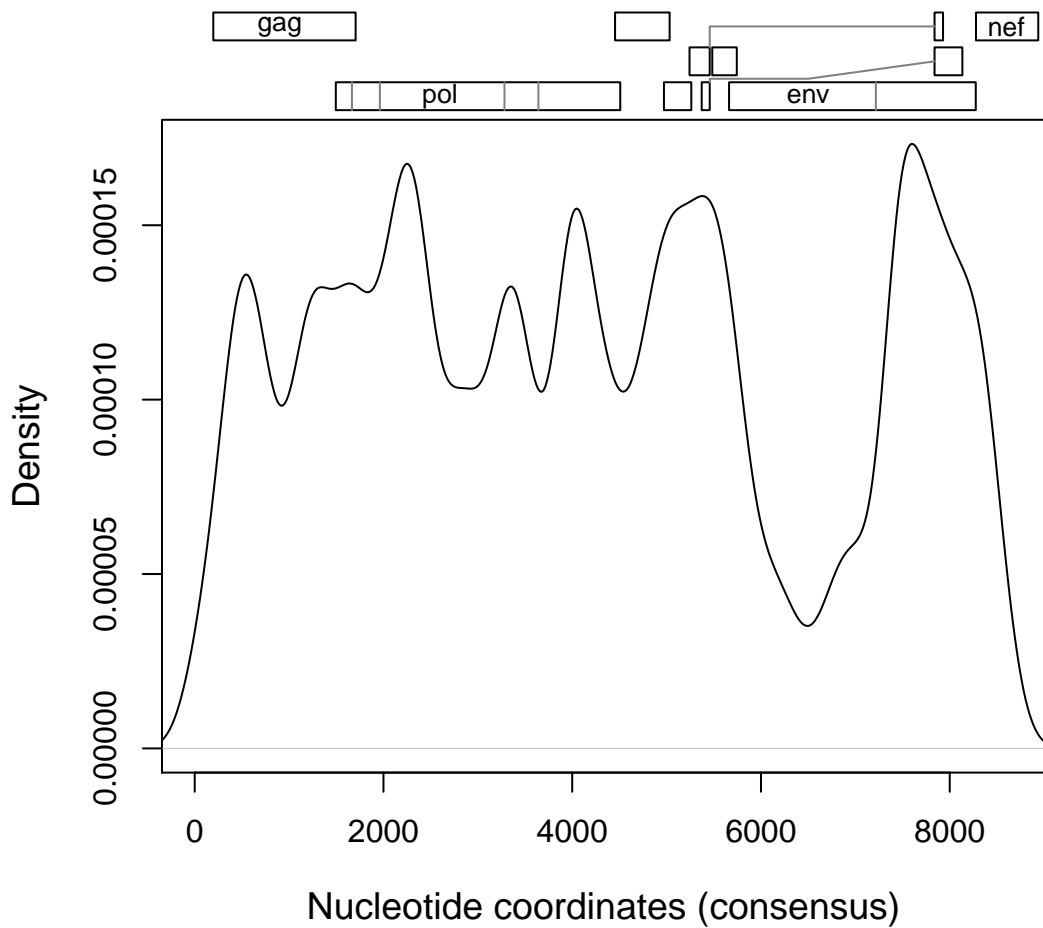

Supplement: Supplementary Figure S6 [file vey039_figure_s6.pdf]

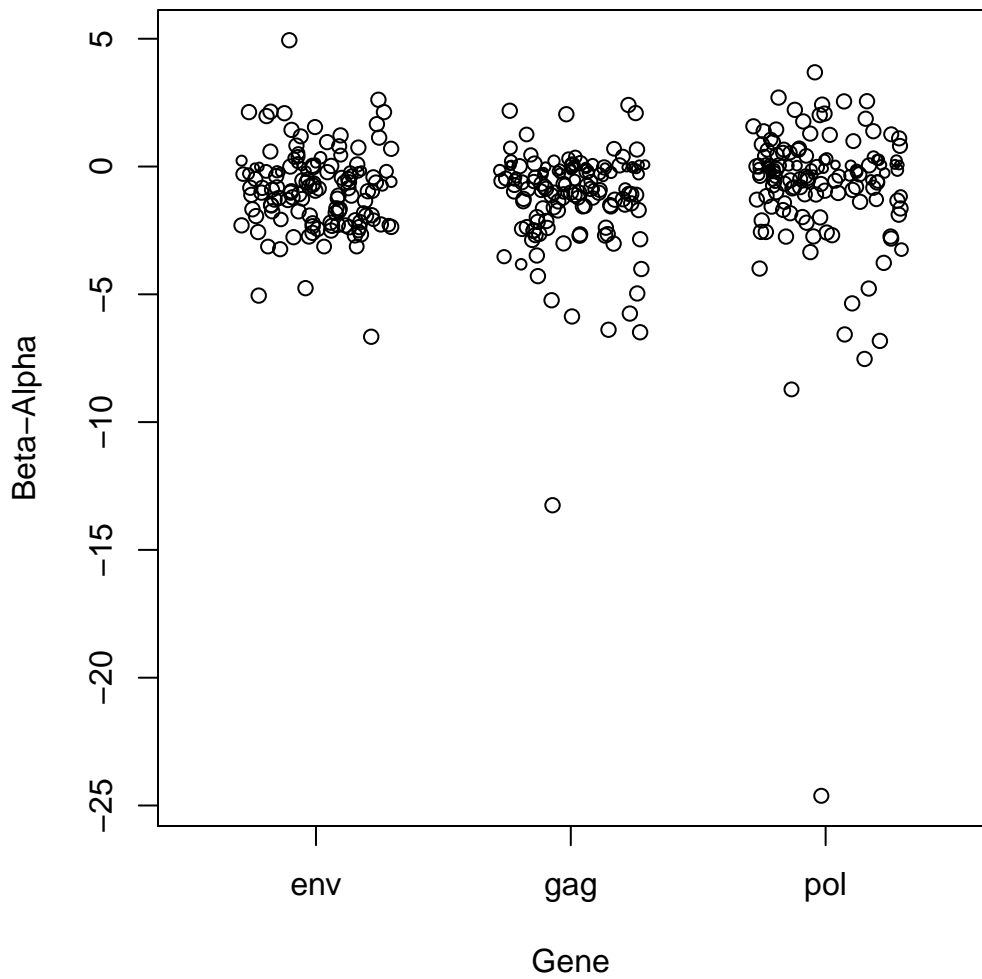

Supplement: Supplementary Figure S7 [file vey039_figure_s7.pdf]

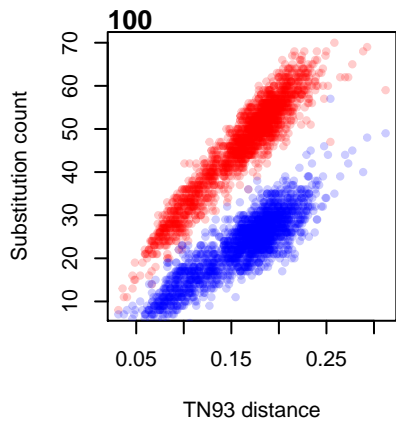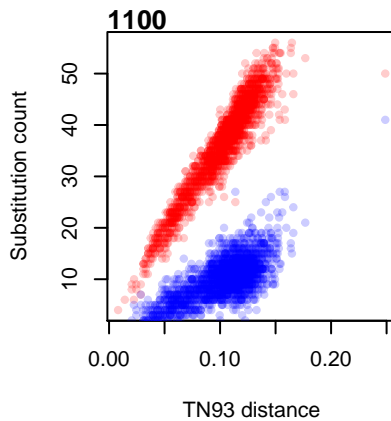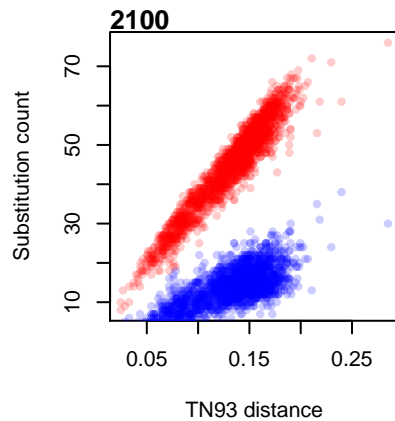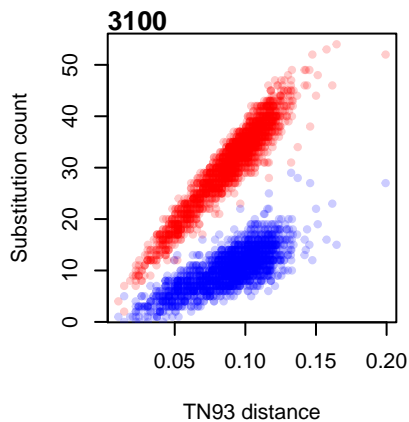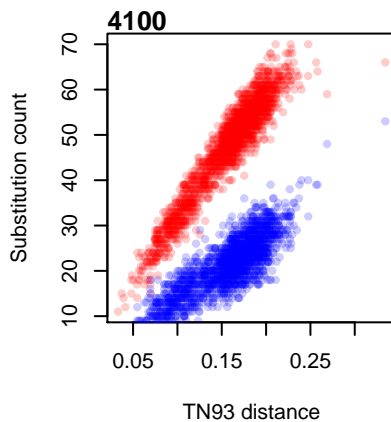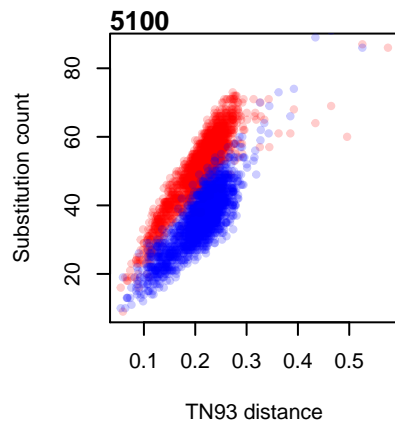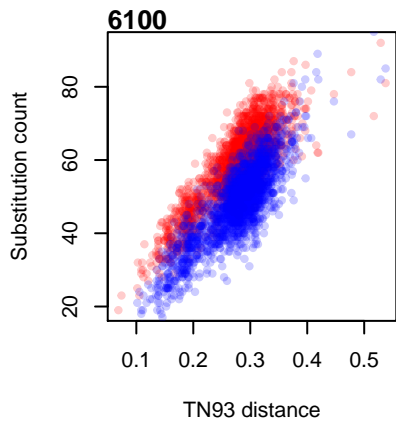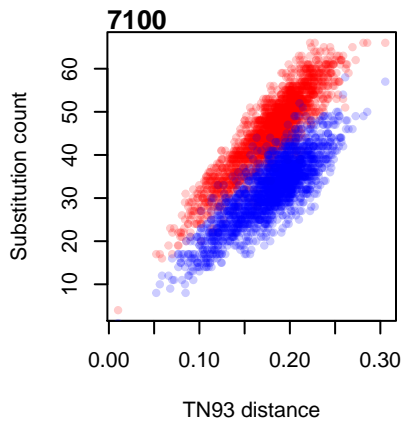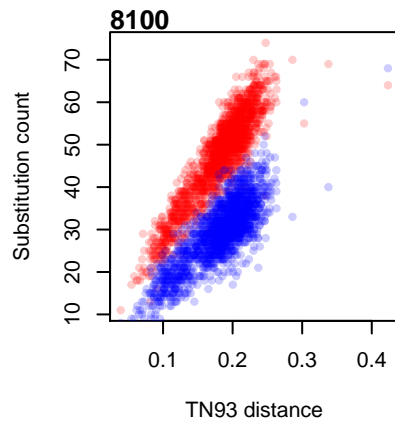

Supplement: Supplementary Figure S8 [file vey039_figure_s8.pdf]
