# Supplementary material for: Evidence for a recombinant origin of HIV-1 Group M from genomic variation
Source: Virus Evol. 2019 Jan 22;5(1):vey039. doi: 10.1093/ve/vey039 (PMC6342232; doi:10.1093/ve/vey039)
Supplement: Supplementary Data [file vey039_supplementary_data.docx]

## Supplementary Figure Legends

Figure 1: **Relationship between the number of HIV-1/M genome sequences and alignment length.** Each box-and-whisker plot summarizes the length of the multiple sequence alignment produced by MAFFT, using 100 random selections of *n* sequences (indicated on horizontal axis) as inputs. The alignment lengths on the vertical axis are measured in nucleotides.

Figure 2: **Relationship between the number of HIV-1/M genome sequences and alignment processing time.** Each box-and-whisker plot summarizes the time required to generate a multiple sequence alignment (MSA, using MAFFT) from a random selection of sequences with a given sample size (x-axis).

Figure 3: **Snapshots of unprocessed and processed HIV-1 group M sequence alignments.** These image files were generated using the program AliView (http://ormbunkar.se/aliview). (top) An alignment of 3,500 HIV-1 genome sequences produced by MAFFT. Note that some individual nucleotides are not visible at this resolution. (bottom) An alignment of all 3,900 genome sequences after pairwise alignment processing.

Figure 4: **Estimated times to the most recent common ancestor (tMRCAs) using Bayesian sampling.** For each window in our alignment of HIV-1/M genome sequences, we randomly sampled up to 10 sequences per year of collection for a total of about 290 sequences (range 284-294). We used BEAST version 1.8.4 to estimate the TRMCA under a flexible Bayesian skyline prior. Each point represents the median tMRCA estimate for a given window from a chain sample of steps, and the grey lines represent the 95% credible interval for each estimate. The x-axis represents the nucleotide coordinate system of the alignment consensus. The green and red lines indicate the breakpoint fragments computed using a piecewise linear regression model selected by AIC.

Figure 5: **Histogram summarizing the number of breakpoints per HIV-1/M genome sequence.** Putative intersubtype recombination breakpoints were identified using an implementation of the RIP algorithm [28] in Python.

Figure 6: **Distribution of putative intersubtype recombination breakpoints.** We used an implementation of the Recombinant Identification Program (RIP) in Python to locate putative breakpoints based on a sliding window analysis of p-distances. The y-axis represents the estimated density function for breakpoint occurrence, for sequences in each window. The x-axis represents the genomic coordinates with each position corresponding to a gene region on the HIV-1 genome map at top of the plot region. A window size of 400 nucleotides and steps size of 5 nucleotides was used.

Figure 7: **Estimates of selection across codon sites in the HIV-1/M major genes.** Each set of points corresponds to site-specific estimates of β−α (the difference between the normalized rates of nonsynonymous and synonymous substitutions) for codons extracted from a window for each of the major HIV-1 genes (*gag*, *pol* and *env*). Selection analyses were performed using the Fast Unconstrained Bayesian Approximation for Inferring Selection (FUBAR) method implemented in *HyPhy* (http://hyphy.org).

Figure 8: **Transition and transversion substitution counts against the Tamura-Nei (TN93) genetic distance in nine window alignments.** The upper label on each plot denotes the leftmost position of each window alignment relative to the consensus genome sequence. For each window alignment, we generated a random permutation of the sequences and then for each pair of sequences, we counted the number of transitions (red) and transversions (blue) and the TN93 distance.
